# Fungi and cercozoa regulate methane-associated prokaryotes in wetland methane emissions

**DOI:** 10.3389/fmicb.2022.1076610

**Published:** 2023-01-06

**Authors:** Linlin Wang, Mingliang Zhao, Xiongfeng Du, Kai Feng, Songsong Gu, Yuqi Zhou, Xingsheng Yang, Zhaojing Zhang, Yingcheng Wang, Zheng Zhang, Qi Zhang, Baohua Xie, Guangxuan Han, Ye Deng

**Affiliations:** ^1^Institute of Marine Science and Technology, Shandong University, Qingdao, China; ^2^CAS Key Laboratory of Coastal Environmental Processes and Ecological Remediation, Yantai Institute of Coastal Zone Research, Chinese Academy of Sciences, Yantai, China; ^3^CAS Key Laboratory for Environmental Biotechnology, Research Center for Eco-Environmental Sciences, Chinese Academy of Sciences (CAS), Beijing, China; ^4^College of Resources and Environment, University of Chinese Academy of Sciences, Beijing, China; ^5^Institute of Soil and Water Resources and Environmental Science, College of Environmental and Resource Sciences, Zhejiang University, Hangzhou, China; ^6^Zhejiang Provincial Key Laboratory of Agricultural Resources and Environment, Zhejiang University, Hangzhou, China; ^7^Collage of Agriculture and Animal Husbandry, Qinghai University, Xining, China; ^8^Yellow River Delta Field Observation and Research Station of Coastal Wetland Ecosystem, Chinese Academy of Sciences, Yantai, China; ^9^Key Laboratory of Coastal Zone Environmental Processes and Ecological Remediation, Yantai Institute of Coastal Zone Research, Chinese Academy of Sciences, Yantai, China

**Keywords:** wetland, inundation level, CH_4_ flux, sediment microbiota, interdomain network

## Abstract

Wetlands are natural sources of methane (CH_4_) emissions, providing the largest contribution to the atmospheric CH_4_ pool. Changes in the ecohydrological environment of coastal salt marshes, especially the surface inundation level, cause instability in the CH_4_ emission levels of coastal ecosystems. Although soil methane-associated microorganisms play key roles in both CH_4_ generation and metabolism, how other microorganisms regulate methane emission and their responses to inundation has not been investigated. Here, we studied the responses of prokaryotic, fungal and cercozoan communities following 5 years of inundation treatments in a wetland experimental site, and molecular ecological networks analysis (MENs) was constructed to characterize the interdomain relationship. The result showed that the degree of inundation significantly altered the CH_4_ emissions, and the abundance of the *pmoA* gene for methanotrophs shifted more significantly than the *mcrA* gene for methanogens, and they both showed significant positive correlations to methane flux. Additionally, we found inundation significantly altered the diversity of the prokaryotic and fungal communities, as well as the composition of key species in interactions within prokaryotic, fungal, and cercozoan communities. Mantel tests indicated that the structure of the three communities showed significant correlations to methane emissions (*p* < 0.05), suggesting that all three microbial communities directly or indirectly contributed to the methane emissions of this ecosystem. Correspondingly, the interdomain networks among microbial communities revealed that methane-associated prokaryotic and cercozoan OTUs were all keystone taxa. Methane-associated OTUs were more likely to interact in pairs and correlated negatively with the fungal and cercozoan communities. In addition, the modules significantly positively correlated with methane flux were affected by environmental stress (i.e., pH) and soil nutrients (i.e., total nitrogen, total phosphorus and organic matter), suggesting that these factors tend to positively regulate methane flux by regulating microbial relationships under inundation. Our findings demonstrated that the inundation altered microbial communities in coastal wetlands, and the fungal and cercozoan communities played vital roles in regulating methane emission through microbial interactions with the methane-associated community.

## 1. Introduction

Methane (CH_4_) has accounted for roughly 30% of global warming since pre-industrial times (Saunois et al., 2016) and, as such, it is generally considered a nonnegligible factor in climate change discussions (Ocko et al., 2021). Wetlands are the largest natural source of CH_4_ emissions, contributing 150–225 Tg CH_4_ per year, and accounting for one-third of total global CH_4_ emissions (Bridgham et al., 2013; Kirschke et al., 2013; Saunois et al., 2016; Dean et al., 2018). In wetlands, water table level controls biogeochemical cycles and has a profound impact on wetland functions (Paschalis et al., 2017). A higher water level can provide a direct barrier to the release of gases from soil or the water column, most likely *via* oxidation as gasses passes through the latter (Peacock et al., 2017), or the inundation level provides an anaerobic environment to inhibit methane aerobic oxidation in the root zone (Li et al., 2018). The process of CH_4_ emission into the atmosphere is ultimately a coordination between CH_4_ production, transportation, and oxidation through the soil-water-plant continuum. Initially, the remains of plant detritus from sedimentary organic matter, a part of this organic matter is released into the atmosphere in the form of CO_2_ again after decomposition by microorganisms, while another part of the organic matter, and inorganic carbon, accumulates in the wetland (Friborg et al., 2003; Fiore-Donno et al., 2020), however, due to the anaerobic environment of wetland water and a large number of microorganisms, fixed carbon in wetland environments will be released into the atmosphere again through respiration or microbial decomposition, mainly as CO_2_ and CH_4_. Hence, CH_4_ fluxes from soils are biologically mediated processes (Shiau et al., 2018). Consequently, focusing on the microbial mechanism behind methane emission under inundation conditions is of great importance.

Microorganisms are linked by strong ecological interactions, and shifts in those interactions could greatly affect ecosystem functioning (de Vries et al., 2013). Previous studies indicated that altering hydrological fluctuation also can reshape soil microbial composition and diversity (Ye et al., 2013; Garssen et al., 2015). Different microbial taxa have displayed divergent responses to inundation across different types of soils or sediments. For example, the structure of the bacterial communities is driven by the hydric dynamics of the infiltration basin, but no such trend was found for fungal communities (Badin et al., 2011). The relative abundance and diversity of methanogenic taxa were much greater in frequently flooded soil than in soil by other means of flooding in the Amazonian floodplain (Hernandez et al., 2019). Unger et al. (2009) found that increased flooding reduced the soil bacteria-fungi ratio by up to 10% in the wetland. Inundation also increased the complexity of prokaryotic communities (Gao et al., 2021), with higher moisture leading to decreased complexity of methanotrophic communities in the Qinghai-Tibetan Plateau (Zhang et al., 2019). Together these studies revealed the diversity and interactions within each soil microbial kingdom responding to water flooding, but far less attention has been paid to interactions among the prokaryotic, fungal, and protist communities under inundation conditions. Therefore, understanding the microbial interactions among different groups or trophic levels along a continuous gradient of inundation is important to assess the functions within coastal wetlands.

Methane flux results from complex interactions between microbes and the environment (le Mer and Roger, 2001). Generally, as the executors for methanogenesis, methanogens can drive the final step of decomposition of anaerobic fermentation end products (i.e., H_2_/CO_2_ and acetate; Bridgham et al., 2013). At present, the extensively studied methanogens belong to the Euryarchaeota, including Methanopyrales, Methanococcales, Methanobacteriales, Methanomicrobiales, Methanosarcinales (Sakai et al., 2011), and Methanocellales (Borrel et al., 2013)/Methanomassiliicoccales (Borrel et al., 2014). The key enzyme of methanogenesis is Methyl Coenzyme M Reductase (MCR), and its encoding gene, *mcrA*, is widely used as a functional gene marker to identify methanogens (Kim et al., 2008). On the other side, aerobic methanotrophs are the most important natural absorbers of CH_4_ emissions from the ecosystem, and the *pmoA* gene encoding the β subunit of the pMMO enzyme is widely used to detect aerobiotic methanotrophs (Freitag et al., 2010; Liebner et al., 2012). Fungi are also vital to many ecosystem processes such as nutrient cycling and decomposition, and they can form direct connections to primary producers (Oliver and Schilling, 2018), while protists can act as a dynamic bond among sediment microorganisms (Xiong et al., 2018), especially with top-down effects on bacterial communities (Zhou et al., 2021). Hence, microbial communities, including prokaryotes, fungi and protists might interact with methane-associated communities to execute disproportionate characteristics in CH_4_ emission processes. Exploration of these interactions may improve our estimates of CH_4_ budgets, resolve CH_4_ dynamics in these environments and improve the predictions of their responses to climate change.

The Yellow River Delta is one of the most active regions of land-ocean interaction in the world (Zhao et al., 2013). As a blue carbon reservoir, it harbors great value in supporting wildlife and is important for climate regulation (Osland et al., 2018). We collected samples from a five-year inundation site experiment in a typical nontidal coastal soil from the Yellow River Delta (Dongying, Shandong, China), which is known for its vital significance in carbon sequestration and climate change (Osland et al., 2016; Feher et al., 2017). Integration of high-throughput DNA sequencing together with multivariate statistical methods can help us understand the divergence among microbial communities in their response to inundation. Three hypotheses were proposed in this study: (i) inundation would tend to alter microbial community compositions and reshape within group microbial interaction networks in this long-term inundation ecosystem; (ii) the changes of fungal and protist communities by inundation, as well as prokaryotes, would lead to the changes of methane emissions; and (iii) changes in sediment properties caused by inundation may contribute to the interactions among these three microbial kingdoms that positively regulate methane emissions.

## 2. Materials and methods

### 2.1. Study site

The study site was located at the Yellow River Delta Ecological Station of Coastal Wetlands (37°45′50″ N, 118°59′24″ E), Chinese Academy of Sciences, Dongying City, in the northeast of Shandong, China. The elevation of the study site was 2.5 m above sea level. The mean annual temperature (MAT) was about 12.9°C and the mean annual precipitation (MAP) was 550–640 mm. The highest percentage of precipitation occurs from June to September. The soil texture is mainly a sandy clay loam with 6.54 g kg^−1^ soil organic matter content at 0–20 cm depth (Zhao M. et al., 2020). At the sampling site, inundation treatment was applied from April to October of each year beginning in 2017. The study area was dominated by common reeds (*Phragmites australis*; Yang et al., 2017). Specifically, the whole surface inundation control test platform included seven inundation level treatments, such as control (no treatment, only natural precipitation), 0 (inundation to soil saturation), 5, 10, 20, 30, and 40 cm (specified surface inundation treatment continued based on soil water saturation) inundation height, respectively. Each treatment had 4 replicate plots, except for the 0 cm inundation treatment which had 3 replicate samples, because one of the samples was missing. Every replicate plot (2 m length × 2 m width × 0.5 m height) was separated by cement barriers at a 40 cm spacing. The height of the cement barriers is 50 cm above and 20 cm underground. The filtered water for each plot was obtained from a nearby lake (originally from the Yellow River which runs into the Bohai Sea). The inundation level was controlled by a tube connected to an opaque plastic water tank located 1.5 meters above the ground surface. When the surface inundation water level falls, water would flow from the tank into the plot until the inundation level reached the original specified level.

### 2.2. Sediment sampling and sediment properties measurement

Sediment cores were collected from each plot from 0 to 20 cm depths on 20th September 2019. After the removal of roots, the roots-removed sediment was gently mixed for homogenization, and then stored at −80°C. Sediment samples were dried using a vacuum freeze-drying machine (18 N; SCIENTZ) for 48 h to prepare for the subsequent experiments. The physical and chemical properties of the sediment were assessed. Sediment pH was measured by mixing dry sediment and distilled water in a 1:2.5 (w/v) ratio. The remaining sediment properties such as organic matter (potassium dichromate oxidation iron salt titration method), total nitrogen (alkaline potassium persulfate oxidation-UV spectrophotometric method), total phosphorus (ammonium molybdate spectrophotometric method), ammonia nitrogen (Nessler’s Reagent Colorimetric Method) and nitrate nitrogen (spectrophotometric method) were all measured at the Institute of Soil Science, Chinese Academy of Sciences (Nanjing, China). CH_4_ flux was measured in a static cylindrical transparent chamber connected to LGR Ultraportable Greenhouse Gas Analyzer (UGGA, Los Gatos Research, Inc., San Jose, USA; Wei et al., 2020). Transparent acrylic plastic base frames (30 cm in diameter, 10, 10, 15, 20, 30, 40 and 50 cm in height) for the seven treatments were installed into the sediment to a 5 cm depth, with 5 cm of the frame emerging above the water surface, a removable middlebox (30 cm in diameter, 100 cm in height) and a removable top box (30 cm in diameter, 100 cm in height) was hermetically placed on the top of the base frame. Two battery-driven fans (8 cm in diameter, 12v) were installed inside the top of each chamber to mix the air in the chamber during sampling. The gas flux was measured from 8 a.m. to 11a.m once a day. For each measurement, chambers were sealed for 3 min.

### 2.3. Sediment DNA extraction amplifications and high-throughput sequencing

After sediment was freeze-dried for at least 48 h (18 N; SCIENTZ), DNA was extracted from 0.5 g of the freeze-dried sediment to extract using the Qiagen DNeasy Power Soil kit. The extracted DNA concentration and quality were assessed with a NanoDrop One Spectrophotometer (Thermo Scientific), then all DNA was stored at −80°C. The primer sets 515F (5′-GTGCCAGCMGCCGCGGTAA-3′) and 806R (5′-GGACTACHVGGGTWTCTAAT-3′) with unique barcodes for each sample were used for amplification of the V4 region of the prokaryotic 16S rRNA gene (Caporaso et al., 2011). The polymerase chain reaction (PCR) amplifications were carried out in a 50 μl of reaction volume, containing 1 μl Phanta Max Super-Fidelity DNA Polymerase (Vazyme, China), 25 μl 2 × Phanta Max Buffer, 1 μl dNTP Mix (10 μM each), 2 μl forward primer, 2 μl reverse primer, 1 μl template DNA (20–30 ng/μL) and 18 μl ddH_2_O. The PCR reaction conditions were as follows: 95°C for 3 min, 30 cycles of 95°C for 10 s, 60°C for 10 s, 72°C for 45 s, and a final extension at 72°C for 5 min. The fungal ITS2 region was amplified using primer sets 5.8S-Fun (5′AACTTTYRRCAAYGGATCWCT-3′) and ITS4-Fun (5′-AGCCTCCGCTTATTGATATGCTTAART-3′; Taylor et al., 2016). The PCRs reactions were carried out in a 50 μl of reaction volumes with 1 μl Phanta Max Super-Fidelity DNA Polymerase (Vazyme, China), 25 μl 2 × Phanta Max Buffer, 1 μl dNTP Mix (10 μM each), 2 μl forward primer, 2 μl reverse primer, 1 μl template DNA (20–30 ng/μl) and ddH_2_O. The reaction conditions were as follows: 95°C for 3 min, 35 cycles of 95°C for 10 s, 52°C for 10 s, 72°C for 45 s, and a final extension at 72°C for 5 min. The V4 hypervariable region of 18S rRNA gene was amplified using a two-step PCR (Fiore-Donno et al., 2018), in the first reaction, the forward primer was a 1:1 mixture of C_615F_Cerco (5′-GTTAAAAAGCTCGTAGTTG-3′) and C_615F_Phyt (5′-GTTAAAARGCTCGTAGTCG-3′), while the reverse primer was C_S963R_Phyt_1st (5′-CAACTTTCGTTCTTGATYAAA-3′), the PCR amplification was carried out in a 10 μl reaction volume containing 0.2 μl Phanta Max Super-Fidelity DNA Polymerase, 5 μl 2 × Phanta Max Buffer, 0.2 μl dNTP Mix (10 μM each), 0.6 μl forward primer, 0.6 μl reverse primer, 1 μl template DNA (20–30 ng/μL) and ddH_2_O (fill the system to 10 μl). The reaction conditions were as follows: 95°C for 3 min, 24 cycles of 95°C for 15 s, 55°C for 15 s, 72°C for 40 s, and a final extension at 72°C for 5 min. The product of the first reaction served as the template of the second reaction after 100-times dilution, the primer set of the second amplification step was C_615F_Cerco_2nd (5’-GTTAAAARGCTCGTAGTYG-3′) and C_S947R_Phyt_2nd (5’-AAGARGAYATCCTTGGTG-3′) with unique barcode sequences, the PCR amplification was carried out in a 50 μl reaction volume containing 1 μl Phanta Max Super-Fidelity DNA Polymerase, 25 μl 2 × Phanta Max Buffer, 1 μl dNTP Mix (10 μM each), 3 μl forward primer, 3 μl reverse primer, 2 μl template DNA (10–20 ng/μL) and ddH_2_O (fill the system to 50 μl). The reaction conditions were as follows: 95°C for 3 min, 10 cycles of 95°C for 15 s, 60 to 50°C for 15 s (decrease 1°C in each cycle), 72°C for 40 s, 25 cycles of 95°C for 15 s, 50°C for 15 s, 72°C for 40 s and a final extension at 72°C for 5 min. All PCR products were purified by gel electrophoresis, and their concentration and quality were measured by NanoDrop spectrophotometer (D2500-02, OMEGA BioTek). Then equal molar amounts of DNA were pooled for library construction and quantified with a Qubit fluorimeter (Invitrogen, Carlsbad, CA). The NovaSeq 6000 platform (Illumina), located at Guangdong Magigene Biotechnology Co. Ltd. (Guangzhou, China), was used to sequence each amplification library. The Illumina sequence reads were deposited in China National Genomics Data Center (CNCB) with accession numbers (PRJCA013006).

### 2.4. Quantification of *mcrA* gene and *pmoA* gene for prokaryotes

To assess the gene copy abundances of *mcrA* and *pmoA* present at different surface inundation levels, real-time quantitative PCR detecting system (qPCR) experiments were performed. The primer sets mlas (GGTGGTGTMGGDTTCACMCARTA) and mcrA-rev1 (CGTTCATBGCRTAGTTNGGRTAGT; Steinberg and Regan, 2008) were used to amplify a fragment of *mcrA* gene and about 469 bps in length. The primers set A189F (GGNGACTGGGACTTCTGG) and mb661r (CCGGMGCAACGTCYTTACC; Knief, 2015) were used to amplify an approximately 470 bps fragment of the *pmoA* gene. Both qPCR experiments were conducted in 20 μl volumes containing 10 μl MonAmp™ SYBR Green qPCR Mix, 0.4 μl forward and reverse primers (10 μM each), 2 μl DNA (5–30 ng), and 7.2 μl ddH_2_O. Three replicates were performed for each sample. The two genes’ cycling conditions were set as follows: 95°C for 30 s, 40 cycles of 95°C for 10 s, 56°C for 10 s and 72°C for 30 s. All reactions were performed in triplicate on a CFX96 Touch Real-Time PCR Detection System (BioRad, California, USA).

### 2.5. Bioinformatics processing and statistical analysis

The raw sequences were processed *via* a public pipeline^1^ (Feng et al., 2017; Zhang et al., 2017). The raw sequence data were sorted after barcode identification, allowing a maximum of one mismatch, and the primers at the ends of sequences were removed. Then pair-end fragments were combined by FLASH (Magoč and Salzberg, 2011). The Btrim program was used to filter out unqualified sequences with a threshold Quality Score > 20 (Kong, 2011). Operational taxonomic units (OTUs) were estimated with a 97% similarity cut-off using UPARSE after chimera filtering and OTU clustering (Edgar, 2013). Taxonomic annotation was conducted using the RDP training set NO.18 (prokaryotes; Wang et al., 2007), UNITE 8.2 (fungi; Nilsson et al., 2019), and PR2 4.14.0 (cercozoa; Guillou et al., 2013; del Campo et al., 2018) databases, respectively. To account for the effects of uneven sequencing depth among samples. we resampled reads in the OTU tables to 50,000, 51,591 and 33,548 sequences for prokaryotes, fungi, and cercozoa, respectively. As the rarefaction curves indicated that the sequencing depth was sufficient, they were then used for the following biodiversity analyses.

We combined control and 0 cm as the low inundation (IL) group, 5, 10, and 20 cm as the moderate inundation (IM) group, while the high inundation (IH) group contained 30, 40 cm, respectively. SPSS Statistics 23.0 (IBM Corporation, Armonk, NY, USA) was used to perform one-way ANOVA analysis to evaluate differences in sediment physiochemical properties, gene copies, and α diversities among different inundation states. In this study, the Shannon index, Pielou evenness, Chao1 and Richness, of prokaryotic, fungal, and cercozoan communities were calculated. Then, for the prokaryotic community, the OTUs belonging to the methanogenic and methanotrophic communities were identified and annotated using the Functional Annotation of the Prokaryotic Taxa (FAPROTAX) database (Louca et al., 2016), and these OTUs were used together in several following analyses. The divergence of community structure was shown using Principal component analysis (PCA) plots. Based on relative abundance, the dominant species of prokaryotic, fungal, and cercozoan communities, methanogenic and methanotrophic OTUs were calculated at the phylum, class, class, order, and order level, respectively. Mantel tests were used to estimate the effect of environmental factors on community structure. Spearman correlation analysis was executed between sediment properties and gene copies while linear regression was performed for the relationships between CH_4_ flux and gene copies. Figures were drawn in R (version 4.0.1) using the ggplot2 package (Murrell, 2009; Wickham, 2011).

### 2.6. Construction of molecular ecological networks under different inundation levels

To investigate the molecular interaction of three microbial communities under different inundation levels, molecular ecological networks (MENs) were constructed. To ensure the reliability of network comparison, according to the PCA analysis results of prokaryotes, we removed the 4 samples that display a large divergence from the other 8 samples in the IM group for the prokaryotic community. Therefore, the sample numbers of the three groups are 7 (IL), 8 (IM), and 8 (IH), respectively. OTUs that were shared by more than 80% of samples were retained to construct the MENs using Spearman’s correlation for prokaryotes, fungi and cercozoa, respectively. For a fair comparison, the correlation thresholds for each group species were kept consistent (prokaryotes = 0.960, fungi = 0.840, cercozoa = 0.890). Finally, the networks were visualized in Cytoscape 3.8.0 and the R package igraph. Then the network topology and global network properties were explored (Deng et al., 2012). Network vulnerability was calculated by the R code provided by Yuan et al. (2021). Robustness calculated the proportion of species remaining in the network after removing random or target nodes (Deng et al., 2012; Montesinos-Navarro et al., 2017).

We further analyzed the networks containing all three microbial communities and methane-associated OTUs to identify their potential functions related to methane emissions. Firstly, we eliminate methanotrophic and methanogenic OTUs from the prokaryotic OTUs. Then the majority of prokaryotes (25), fungi (9), methanotrophs (3), methanogens (3), and cercozoa (9) was performed, and used to calculate the appropriate threshold (0.85) based on the RMT method. In each network, the roles of individual nodes were estimated by two topological parameters: the within-module connectivity *Zi*, which quantified to what extent a node connected to other nodes in its own module, another is the among module connectivity *Pi*, which quantified how well the node connected to different modules. The nodes with either a high value of *Zi* or *Pi* were defined as keystone taxa, including module hubs (*Zi* > 0.25, *Pi ≤* 0.62; critical to its own module coherence), connector hubs (*Zi* < 0.25, *Pi* > 0.62; connect modules together and important to network coherence), network hubs (*Zi* > 0.25, *Pi* > 0.62; vital to both network and its own module coherence), all other nodes are peripherals nodes (le Mer and Roger, 2001). The network modules detection was performed using fast greedy modularity optimization (Deng et al., 2012). For network modules, the module eigengene could summarize the closely connected members within a module (de Menezes et al., 2015). The singular value decomposition of the module expression matrix was used to represent the module eigengene networks (Alter et al., 2000). The module eigengene of the module was defined as the first principal component of the standardized module expression data (Langfelder and Horvath, 2007). In addition, the correlations between module-based eigenvalues and sediment properties were calculated based on Pearson algorithm (Deng et al., 2012; Feng et al., 2019). Both networks and modules were visualized in Cytoscape 3.8.0. All of the above methods and statistical tools for the networks were executed on the Inter-Domain Ecological Network Analysis Pipeline (iNAP)^2^ (Feng et al., 2022).

## 3. Result

### 3.1. Physicochemical properties and CH_4_ flux shifted under different levels of inundation

**Figure 1:**
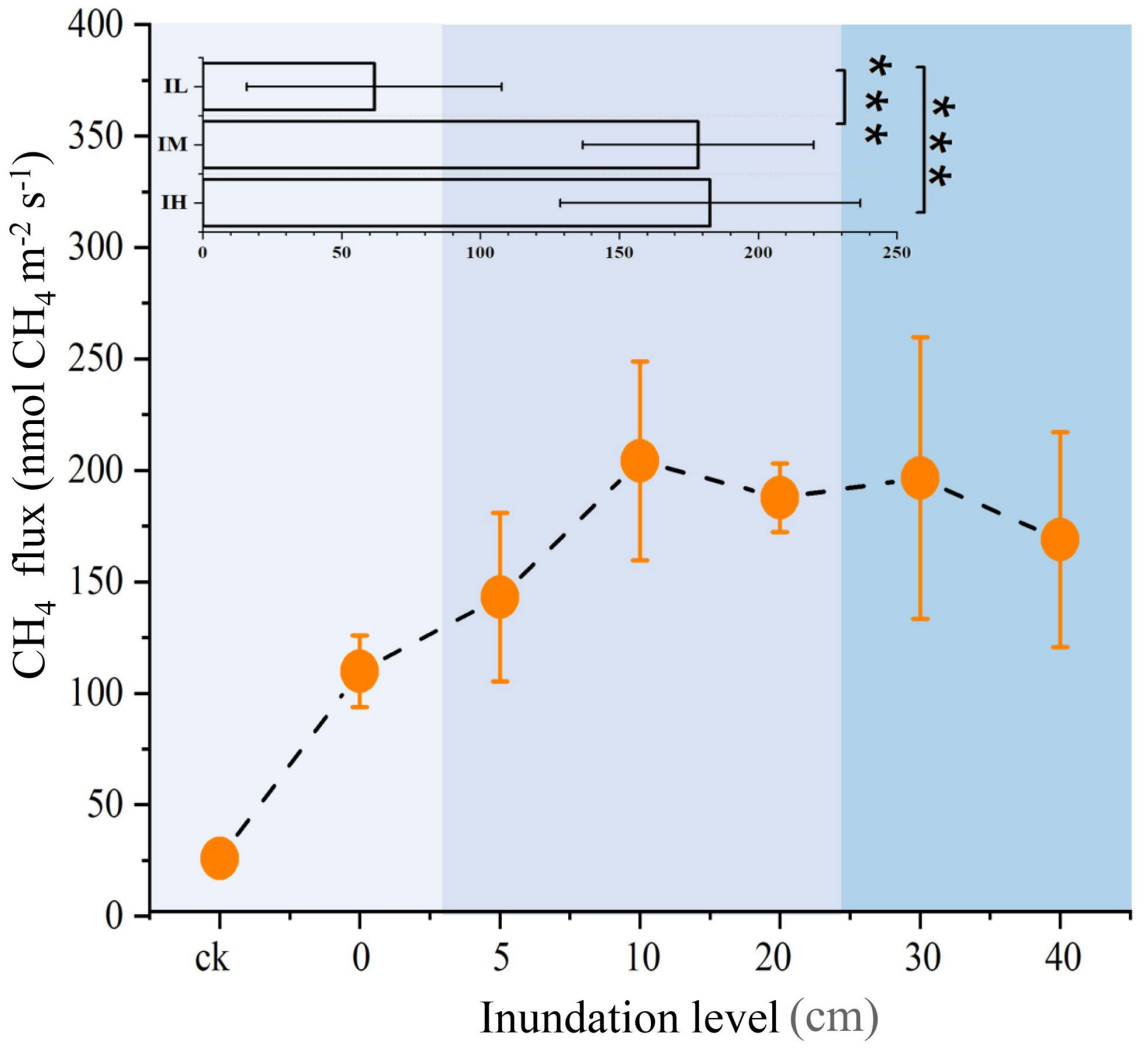
Variations in CH_4_ fluxes at different inundation depths (means ± SE, *n* = 27). In insets, inundation depths in soil were subjected to three treatments: Soils were exposed to low inundation (IL, *n* = 7), moderate inundation (IM, *n* = 12) and high inundation (IH, *n* = 8). One-way analysis of variance (ANOVA) of three groups was performed. For all panels, the extent of blue backgrounds indicates the three groups. ***p < 0.001.

Sediment CH_4_ flux and physicochemical properties were altered by long-term inundation. As the inundation level rose, CH_4_ flux also gradually rose initially, reaching a maximum at 10 cm inundation, but then decreased as the inundation level continued to rise (Figure 1). On average, the values of CH_4_ flux increased by 188 and 195% under the moderate inundation (IM) and the high inundation (IH) groups, respectively, than in the low inundation group (IL; *p* < 0.05; Supplementary Table S1). Among the physicochemical properties, dissolved oxygen and sediment pH were significantly lower in IM than in IL (*p* < 0.05). In addition, total sediment phosphate was 33% higher in IH than in IL (*p* < 0.05). Nitrate nitrogen decreased with inundation levels (*p* < 0.05). However, there were no detectable variations for total nitrate, ammonia nitrogen, organic matter, or salinity among the different inundation groups (Supplementary Table S1). These results suggested that inundation significantly altered the CH_4_ flux and several physicochemical properties.

### 3.2. Inundation changed the abundance of *mcrA* and *pmoA* genes in soil

**Figure 2:**
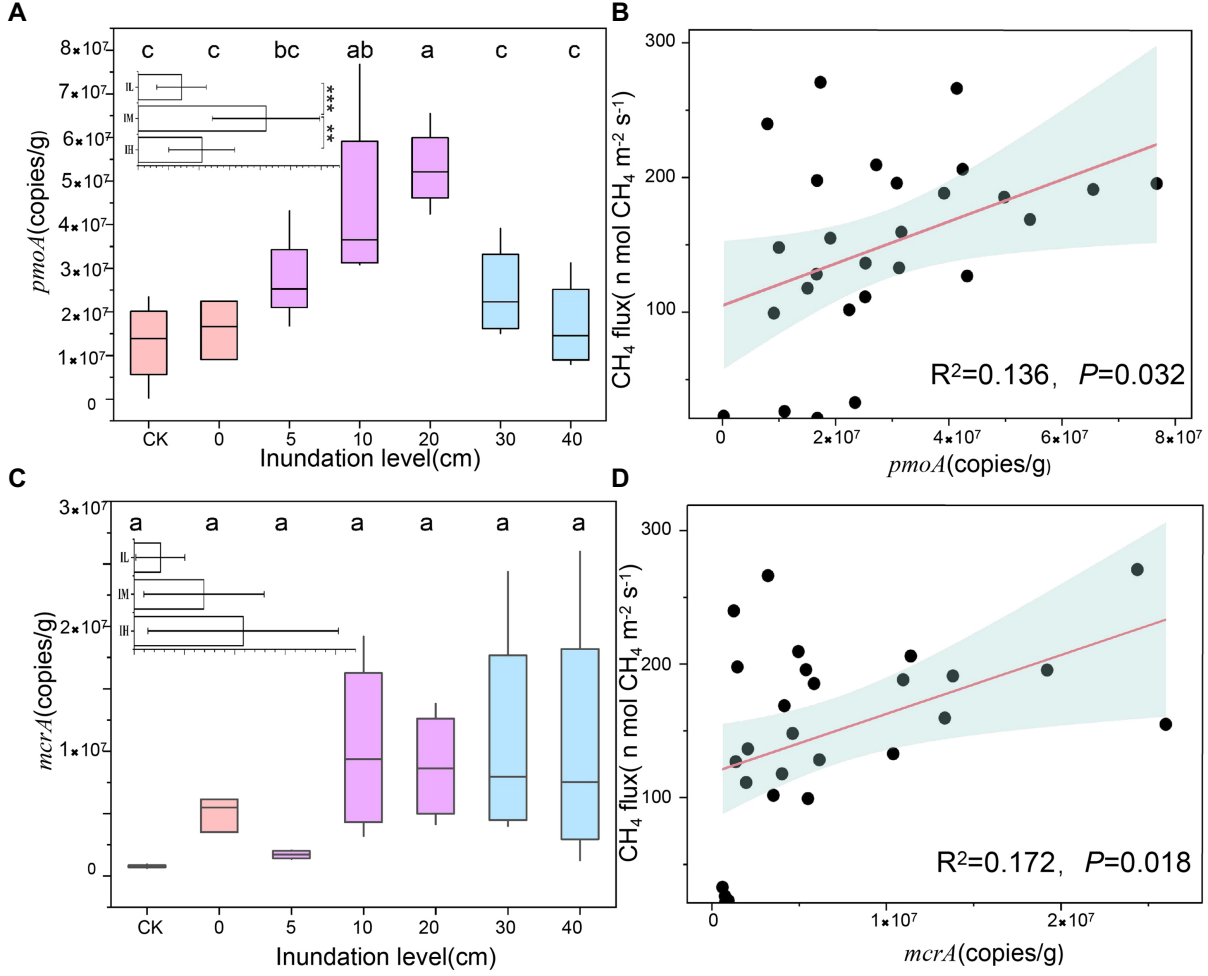
Variations in gene copies at different inundation depth and its relationship with CH_4_. **(A)** Variations in *pmoA* gene copies at different inundation depths (means ± SE, *n* = 27). In insets, inundation levels in soil were subjected to the three treatments: Soils were exposed to low inundation (IL), moderate inundation (IM) and high inundation (IH), IL (*n* = 7), IM (*n* = 12), IH (*n* = 8), one-way analysis of variance (ANOVA) of the three groups was performed. **(B)** Relationships between *pmoA* gene copies and CH_4_, The pink lines are fitted regressions, and the light blue-shaded areas around the regression lines are 95% confidence. **(C)** Variations in *mcrA* gene copies at different inundation levels (means ± SE, *n* = 27). **(D)** Relationships between *mcrA* gene copies and CH_4_ flux. Values with superscript letters a, b, c and are significantly different across columns (*p* < 0.05).

Functional genes of the two methane-associated cycles, i.e., *pmoA* and *mcrA* showed distinct tendencies with inundation levels (Figure 2). *pmoA* gene copies increased slightly, peaking at the inundation level of 20 cm, subsequently decreasing sharply (5.3 ± 0.9 × 10^7^ g^−1^ to 1.3 ± 0.9 × 10^7^ g^−1^), showing a significant difference under 30 cm and 40 cm inundations (*p* < 0.01) compared to 20 cm inundation (Figure 2A). However, the *mcrA* gene copies displayed no distinct trend under different inundation levels (Figure 2C). A significant linear relationship was found between CH_4_ flux and *pmoA* gene copies (*R*^2^ = 0.136, *p* = 0.032; Figure 2B) and *mcrA* gene copies (*R*^2^ = 0.172, *p* = 0.018; Figure 2D). Additionally, the *pmoA* gene quantity in soil was significantly positively correlated with oxygen content (Supplementary Table S2). The above results indicated that the inundation level could change the abundance of *pmoA* genes more significantly than *mcrA* genes, and the greater inundation levels may accelerate methane utilization rather than methane generation.

### 3.3. Inundation shifted the composition and structure of soil microbial communities

To estimate the α diversity of microbial communities under different levels of inundation, several indexes were calculated, including the Shannon index, Pielou evenness, Chao1 and Richness index. In the prokaryotic community, the Shannon (*p* = 0.046) and Pielou evenness (*p* = 0.044) indices significantly decreased in the IM group compared with the IL, while the shift of these two indices between the IL and IH groups was not significant (Supplementary Figures S1a–d), no significant variation among these indices was found in the fungal community (Supplementary Figures S1b–h). In the cercozoan community, Chao 1 increased in the IH group compared with IM group (Supplementary Figures S1i–l). Next, using FARPROTAX, 67 methanogenic OTUs and 23 methanotrophic OTUs were identified from the prokaryotic community. Accordingly, we also performed α diversity analysis on these OTUs. Chao 1 and observed richness were significantly increased in the methanotrophic group from IL to IM, and Chao 1 was significantly decreased from IM to IH (Supplementary Figures S1m–p), however, α diversity indices of methanogens did not show any significant difference among the three inundation groups (Supplementary Figures S1q–t).

The β diversity of prokaryotes and fungi was remarkably divergent among the three groups based on the multiple dissimilarity tests, but for the cercozoan community, there was only a significant difference between the IL and IH groups. In the methanogenic community, there was a significant difference between the IL and IM groups, while no significant differences were observed in the methanotrophic community (Supplementary Table S3). Moreover, PCA analysis of the microbial communities showed a distinct separation between the IL and IH groups, while the IM community was only partially separated from the others (Supplementary Figures S2a–e). The composition of microbial communities also shifted under inundation (Supplementary Figures S2f–j). Additionally, to discern the associations between soil variables and the structure of all microbial communities. Mantel tests based on Jaccard distance were performed. The results indicated that the CH_4_ flux was significantly correlated with the prokaryotic (*p* = 0.002), fungal (*p* = 0.001), and cercozoan (*p* = 0.001) communities (Table 1).

**Table 1:** Mantel tests for the correlation between environmental variables and community structure in prokaryotic, fungal, cercozoan communities based on Jaccard distance.

		TN (mg/kg)	TP (mg/kg)	Ammonium (mg/kg)	Nitrate (mg/kg)	DOM (%)	pH	Salinity (ppt)	Oxygen (vol%)	CH_4_ flux (n mol CH_4_ m^−2^ s^−1^)
Prokaryotes	*r*	−0.1704	−0.1295	0.004	0.0535	0.057	0.0811	0.0085	0.2299	0.5425
	*p*	0.963	0.874	0.444	0.285	0.255	0.187	0.431	**0.006**	**0.001**
Fungi	*r*	−0.0942	−0.1401	0.101	0.1992	0.0999	0.0933	0.0317	0.1414	0.3909
	*p*	0.81	0.908	0.056	**0.013**	0.114	0.129	0.335	**0.025**	**0.001**
Cercozoa	*r*	−0.0935	−0.0451	0.0333	0.0423	−0.0205	0.0705	0.0646	0.2218	0.4418
	*p*	0.771	0.648	0.286	0.338	0.604	0.222	0.22	**0.003**	**0.001**

Significantly (*p* < 0.05) related environment variables are shown in bold.

The functional divergence observed in the prokaryotic community, especially, the abundances of methanogenic and methanotrophic microorganisms, increased with inundation depth (Supplementary Figure S3a). Among methanogens, the hydrogenotrophic methanogenesis pathway dominated, especially the hydrogenotrophic pathway *via* the CO_2_-reducing pathways, while relative abundances of the acetoclastic and methylotrophic methanogenesis pathways were lower (Supplementary Figure S3b). These results suggested that inundation played an important role in shaping the microbial communities, and that there were significantly positive correlations between microbial communities and methane flux.

### 3.4. The shift of co-occurrence networks under different levels of inundation

Co-occurrence networks for each microbial group (prokaryotic, fungal and cercozoan communities) were explored to characterize the shift of potential interactions along the inundation gradient (Supplementary Figure S4), and their global topological properties were measured (Supplementary Table S4). For the prokaryotic community, the number of nodes decreased in IM and then increased sharply in IH, with similar patterns observed for the fungal and cercozoan communities. The number of edges within the networks showed a similar trend as the nodes. Network complexity decreased according to changes in average path distance (GD) and clustering coefficient (avgCC). The vulnerability of prokaryotes and cercozoa decreased at first before increasing with the increase of inundation levels, while the vulnerability of the fungal community increased initially and then decreased with the increase of inundation levels (Supplementary Table S4). The keystone taxa including modules hubs (Zi > 0.25, Pi ≤0.62), and connectors (Zi ≤ 0.25, Pi >0.62) were identified (Supplementary Figure S4). For prokaryotic networks, the module hubs changed from Planctomycetes and Proteobacteria to Actinobacteria, Acidobacteria, Planctomycetes, Chloroflexi and Proteobacteria, while the connector hubs shifted from many different taxa to Rhodothermaeota, Actinobacteria, Acidobacteria, and Chloroflexi. Initially, there were no module hubs in the fungal networks, and then taxa such as Sordariomycetes, Pezizomycetes, Dothideomycetes became module hubs, and Saccharomycetes and Sordariomycetes became the connectors. For cercozoan networks, only the connectors shifted from Endomyxa to Filosa-Sarcomonadea and Endomyxa-Phytomyxea (Supplementary Figure S4). Therefore, these results indicated that the vulnerability of the microbiome changed in different ways, and the keystone species in co-occurrence networks for prokaryotic, fungal and cercozoan communities shifted with increasing inundation levels.

### 3.5. Intra-kingdom ecological association networks under inundation

**Figure 3:**
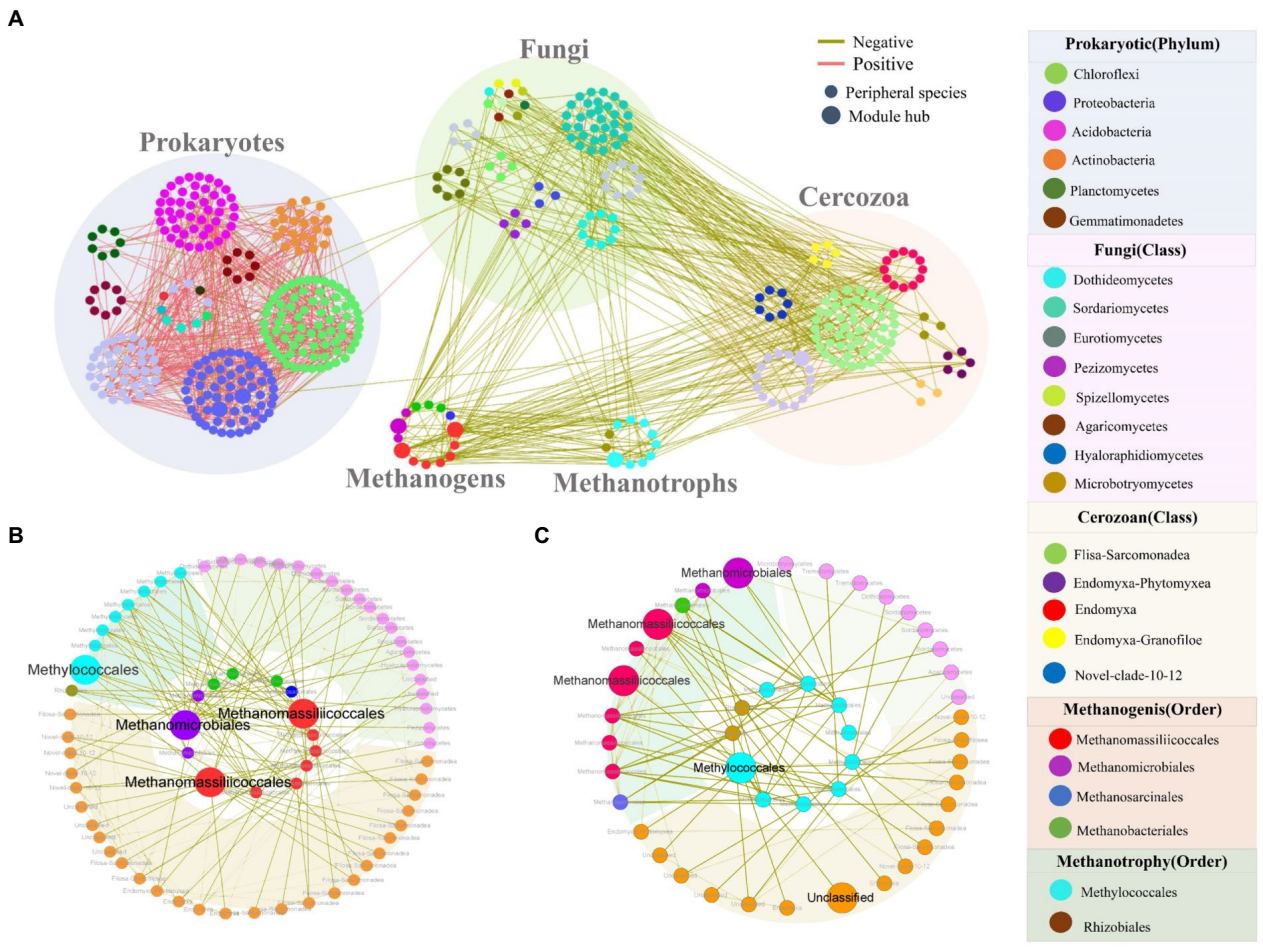
Intertrophic networks among five communities and two subnetworks relative to methanotrophic OTUs and methanogenic OTUs. **(A)** Intertrophic networks of prokaryotic, fungal, cercozoan communities, methanotrophic OTUs and methanogenic OTUs under inundation. Node color indicates different taxonomic. Node size is proportional to network role, with larger nodes being module hubs. Red and green links represent positive and negative interactions, respectively. **(B)** Subnetworks showing the OTUs related to methanogenic OTUs, inner circle are methanogenic OTUs while outer circles are their connected OTUs. **(C)** Subnetworks showing the OTUs relative to methanotrophic OTUs, inner circle are methanotrophic OTUs while outer circles are the connected OTUs. The background colors represent different communities: fungi (purple), cercozoa (pink), methanotrophic OTUs and methanogenic OTUs (green).

Due to all prokaryotic, fungal and cercozoan communities being significantly correlated with methane flux (Table 1), we constructed an intra-kingdom ecological network to discern the potential contributions of fungi and cercozoa to methane-associated cycles. The integrated network contained 478 nodes, including 100 cercozoa, 269 prokaryotes, 84 fungi, 11 methanotrophs, and 14 methanogens (Figure 3A). Interestingly, methanogenic and methanotrophic taxa displayed negative relationships to fungal and cercozoan OTUs, but no connections with other prokaryotic taxa. Numerous positive relationships were observed among the prokaryotic taxa not involved in methanogens or methanotrophs (Figure 3A). More nodes were connected to methanogenic taxa (Figure 3B) than to methanotrophic taxa (Figure 3C). Only seven module hubs were identified as keystone nodes in this network, i.e., two nodes from prokaryotic taxa (Proteobacteria), three from methanogenic taxa (two nodes annotated Methanomassiliicoccales, one node annotated Methanomicrobiales), one from methanotrophic taxa (Methylococcales), one from cercozoan taxa, which was annotated Unclassified at class level. In methanogenic taxa, two of the three module hubs annotated Methanomassiliicoccales were closely related to the methanotrophic taxa and cercozoan taxa, while the taxa closely related toward the Methanomicrobiales were methanotrophic taxa and fungal taxa (Figures 3A,B). For methanotrophic taxa, the module hub Methylococcales was connected intimately with methanogenic taxa and cercozoan taxa (Figures 3A,C).

**Figure 4:**
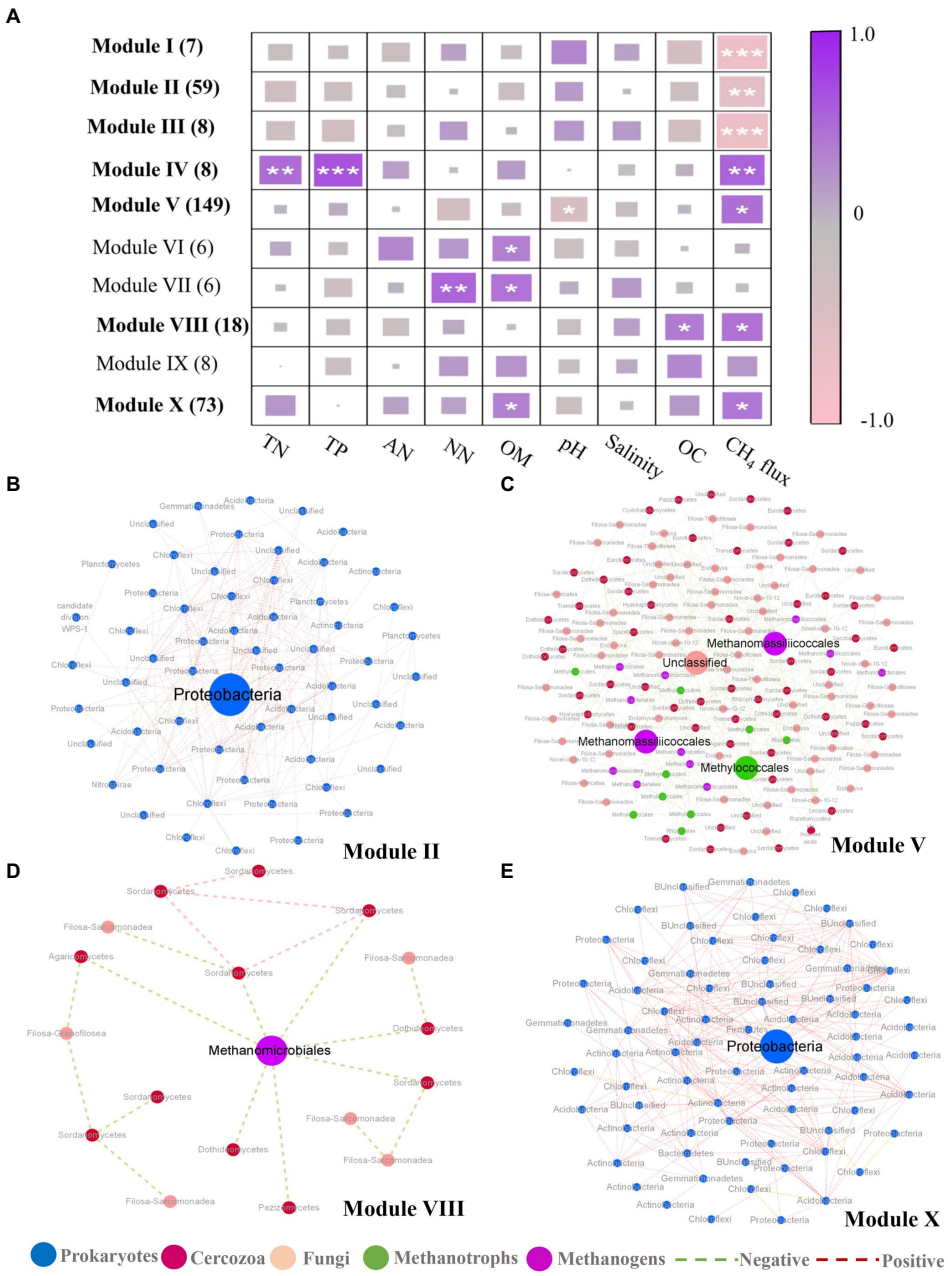
Correlation between module eigengenes and soil properties and display of related modules. **(A)** Correlation coefficients between module eigengenes and soil properties together with ecosystem methane (CH_4_) under inundation treatments. The numbers in parentheses indicate the number of nodes observed in each module. Module eigengenes significantly related to CH_4_ are in bold. Asterisks denote the significant relationships (*p < 0.05, **p < 0.01 and ***p < 0.001). TN, total nitrogen; TP, total phosphorus; AN, Ammonia nitrogen; NN, Nitrate nitrogen; OC, Oxygen content. **(B–E)** Representative modules that were significantly related to CH_4_ under inundation. In each module, node size is proportional to the network role, with larger nodes representing module hubs. Node color represents taxonomic groups. Prokaryotes (blue), cercozoa (red), fungi (pink), methanotrophic OTUs (green) and methanogenic OTUs (purple).

In addition to the discovery of key nodes in the network, the specific clustering modules were examined to find significant module-trait related to CH_4_ flux. Modules I-III showed significant negative correlations with CH_4_ flux, whereas Modules IV, V, VIII, and X showed an inconsistent trend (Figure 4A; Supplementary Table S5), among these modules, modules I, III and IV only contain prokaryotic OTUs (Supplementary Table S5). Four subnetworks, consisting of modules II, V, VIII, and X, respectively, were further extracted due to the presence of module hubs. The modules negatively correlated with methane were all composed of prokaryotic OTUs (Supplementary Table S5). Of these modules, module II had a module hub annotated as Proteobacteria (Methyloceanibacter at the order level) that had many positive correlations (Figure 4B). In modules that were positively correlated with methane fluxes, there were 4 module hubs in module V (Figure 4C), two from methanogenic taxa (Methanomassiliicoccales), one from methanotrophic taxa (Methylococcales), and one from cercozoan taxa. Methanomassiliicoccales possessed more connections with methanotrophic taxa (mainly annotated as Methylococcales) and cercozoan taxa (mainly annotated Filosa-Sarcomonadea; Supplementary Figures S5b,c). The Methylococcales module hub was related more to cercozoan taxa (Fliso-Sarcomonade; Supplementary Figure S5a). The cercozoan module hub was not connected to any methanogenic taxa, but it is noteworthy that it was significantly related to the module hubs annotated Methylococcales (Supplementary Figure S5d). Module VIII had one module hub, Methanomicrobiales, which was from the methanogens, and it was more tightly bound to fungi than cercozoa (Figure 4D). In module X, the module hub belonged to the Proteobacteria, and the module contained positive relationships (Figure 4E). These results suggested that cercozoa and fungi had close associations with methane-associated prokaryotes.

## 4. Discussion

Atmospheric methane is an important topic in climate change discussions, and its most significant source is all kinds of wetland ecosystems (Saunois et al., 2016). Microorganisms act as both the generators (methanogens) and consumers (methanotrophs) of methane, and thus they make a significant contribution to climate change. Some non-methanogenic/methanotrophic microorganisms may play important roles in the process of methane emission. For example, several unicellular protists participate in the flow of carbon mediated by food chains and microbial loops as consumers (Adl et al., 2019). Based on the respiration of their massive amounts of hyphal material, several fungi are a driving force in the biological component of the terrestrial carbon cycle (Barron, 2003). However, fungi and protists associated with methane emissions have previously only been studied in rumen studies of cattle (Lopez-Garcia et al., 2022), and less attention has been paid to their role in natural ecosystems. In a natural ecosystem, microorganisms associate with each other through complex interactions (Zhang et al., 2018; Zhao Y. et al., 2020; Qian et al., 2021), and exploring these interactions through co-occurrence networks (Fan et al., 2018) could help us to better understand ecosystem function and maintenance mechanisms (Kobayashi and Crouch, 2009; van Overbeek and Saikkonen, 2016; Deveau et al., 2018). Here, we systematically investigated the cross-trophic networks among prokaryotes, fungi, protists and methane-associated communities in a gradient of simulated inundation conditions.

The shift in microbial community diversity, structure, and composition showed an inconsistent tendency along divergent inundation levels among different microbial groups. Previous studies have proposed that inundation can adjust the diversity and composition of microbial communities (Rinklebe and Langer, 2006; Goldman et al., 2017; Shiau et al., 2018; Yang et al., 2019; de Jong et al., 2020). Here we compared the microbial diversity (Shannon index, Pelious evenness, Chao1 index, Richness), structure, and composition among IL (CK, 0 cm), IM (5–20 cm), IH (30–40 cm) groups (Supplementary Figures S1, S2). Our results demonstrated that prokaryotic Pelious evenness decreased in IM as compared to IL, which was inconsistent with observations from mangrove peat soil under inundation (Chambers et al., 2016). Badin et al. (2011) observed the fungal biomass was less driven by moisture content, similar to the observations in this study, while previous studies in three kinds of the floodplain showed that fungi had low survival rates in chronically submerged soil (Rinklebe and Langer, 2006). The cercozoan diversity in IH was higher than IM. The methanotrophic community showed a parallel tendency with the whole prokaryotic community. Methanogenic diversity showed no divergence, even though previous studies indicated that methanogenic community would be affected by flooding (Hernández et al., 2019; Shen et al., 2022). Except for these changes in methane-related microbes, methane-related genes (*mcrA* and *pmoA*) showed a significant positive correlation with methane flux under inundation conditions. First, the substrate of methane generation was produced, then the *mcrA* gene could express to produce methane based on this substrate, therefore, the copy numbers of *mcrA* gene are consistent with the methane flux (Steinberg and Regan, 2008). The positive correlation between *pmoA* gene copies (Knief, 2015) and methane flux may be due to that methane generation may directly affect the expression of the *pmoA* gene in an inundation environment, as soon as methane is created, it is rapidly utilized. These consistent or inconsistent tendencies in prokaryotic, fungal, and protistan diversity indicated that coastal soils could be susceptible and the divergence in microbial diversity did not linearly correlate with the inundation level gradient. In addition, the changes of methane-related microbes and methane-related gene expression showed a unique pattern under inundation condition.

Except for biodiversity, the co-occurrence networks within each microbial community along the inundation gradient were altered. Within the intradomain networks (Supplementary Figure S4; Supplementary Table S4), the number of edges and nodes first decreased and then increased along the gradient of inundation level, and this trend is consistent with the change in richness values and Chao 1. The phenomenon may be due to a partial plant stomatal closure under water with lower photosynthesis activity in the submerged leaves, when the inundation reaches a certain level (Schedlbauer et al., 2010; Han et al., 2015). Thus, an anaerobic environment is formed, resulting in the extinction of aerobic microorganisms. However, if the inundation level rises again to a higher level, more plant shoots and leaves are submerged, and the increase in organic residues provides resources for microorganisms, which may buffer the adverse effects of the anaerobic environment on the microbes. Inundation resulted in a slight increase in the vulnerability of the prokaryotic community, but a more pronounced change in the fungal and cercozoan communities, possibly due to their inherently low diversity. Inundation causes water to infiltrate into the soil pore space, altering the distribution of nutrient content. Also, the flowing water bodies create difficulties for microbial colonization, further leading to heterogeneity and irregularity in the distribution of these microorganisms in the soil. As a result, their species interactions become more susceptible to external influences under the stressful environments of inundation. Except for it, the robustness of the network was calculated (Supplementary Figure S6), as for prokaryotic communities, the robustness increased significantly at high inundation levels, but the robustness of fungal communities and cercozoan communities did not change significantly (Deng et al., 2012; Montesinos-Navarro et al., 2017). The result suggested that prokaryotic communities are more likely to remain stable under high inundation conditions. In addition, keystone species also shifted greatly in the co-occurrence network along different inundation levels. Similar results were reported in Gao’s study, which revealed that increasing water content can significantly decrease the relative abundance of keystone species (Gao et al., 2021). Keystone species such as connectors or module hubs are considered to play critical roles in network structure maintenance (Deng et al., 2012; Ma et al., 2016), and this shift also demonstrated the sensitivity of molecular interactions to inundation as well. Taken together, inundation can make the interaction between microorganisms fragile, especially in fungal and cercozoan communities.

In this study, the methane-associated communities may show a preference for interaction under inundation, as they may be more likely to negatively interact with the cercozoan and fungal communities (Figure 3). This inverse correlation was inconsistent with previous studies in the rumen of animals, where the metabolites of anaerobic fungi can be used by methanogens to co-exist (Wood et al., 1986; Nakashimada et al., 2000). A possible explanation for this divergence may be due to microorganisms from different sources showing a diversity of birth environment preference and metabolic mode. Hydrogen generated by ciliated protozoa can be utilized by methanogens to synthesize methane for methanogenic taxa, but it is important to note that not all protozoa are the same (Newbold et al., 1995; Ranilla et al., 2007). For example, holotrich protozoa play a disproportionate role in supporting methanogenesis by competing with methanogens for nutrients (Newbold et al., 2015). Cercozoa is known to feed on bacteria, fungi, and even some eukaryotes (Dumack et al., 2020), and thus another factor may be that the predation effect of cercozoa on microbiota is much stronger than its synergistic effect on methanogens in an inundation environment. The negative correlation between methanogenic communities and fungi, cercozoa may result from resource competition and predation. But for methanotrophic taxa, it is likely due to apparent competition (Holt, 1977) and predation, respectively. Because of the priority effects provided by negative correlation, late-arriving species could not grow because of the effects of early arriving species, the competition or predation can stabilize the fluctuation of community with disturbance and promote the stability of the network (Coyte et al., 2015; de Vries et al., 2018). Conventionally, methanogens could cooperate with the fermenting bacteria and syntrophic bacteria to finish the typical anaerobic syntrophic oxidation (McInerney et al., 1979), however, in this study, methane-related microbes were more likely to interact with the cercozoa and fungi, the reasons may as follows: the function and composition of syntrophic bacteria and fermenting bacteria may become fragile due to the influence of water osmotic pressure (Badin et al., 2011), and their potential ability to degrade substrates became weakened. In addition, hydrological changes may affect the interactions among microorganisms (Unger et al., 2009), to stabilize the whole community, methane-related microbes tend to reinforce negative interactions with cercozoa and fungi among many microorganisms rather than the positive interactions with prokaryotes (Coyte et al., 2015; de Vries et al., 2018). Therefore, the preference and negative correlation of methane-associated microorganisms with cercozoa and fungi may be a special way to maintain a stable species interaction state in an inundation environment.

Among several methane-related modules, environmental factors tend to affect the modules positively correlated with CH_4_ flux, and pH may be an important environmental factor in regulating modules. Among the modules negatively correlated with methane, the Alphaproteobacterial OTU annotated as Methyloceanibacter (genus) was one of the module hubs in module II (Figure 4B). Marine methylotrophs are vitally important in the global carbon cycle as they metabolize one-carbon compounds. One of the newly isolated Methyloceanibacter species from the North Sea was found to be capable of oxidizing methane as the sole source of carbon and energy by solely using a soluble methane monooxygenase (Takeuchi et al., 2014; Vekeman et al., 2016). Therefore, Methyloceanibacter is crucial for suppressing methane production under inundation. In module V (Figure 4B), one of the four module hubs was a cercozoan OTU (Figure 4C), which indicated that cercozoa may be a nonnegligible component of the soil microbiome under inundation. Previous studies show that protists can act as dynamic bonds among soil microorganisms (Xiong et al., 2018; Asiloglu et al., 2021). It was interesting that there was a significant negative correlation between this cercozoan OTU and a Methylococcales module hub, the negative correlation may contribute to the positive association of the whole module with methane emissions. The other two module hubs were Methanomassiliicoccales and Methanomicrobiales, the form of which is a new methanogenic archaea order, belonging to the Euryarchaeota, present in marine and lake habitats (Söllinger et al., 2016), and can use external H_2_ to reduce methyl compounds for the production of methane (Borrel et al., 2014). Methanomicrobiales are widely found in mangrove sediments, which is one of the more abundant Euryarchaeotal methanogenic orders (Li et al., 2012; Zhou et al., 2014). In modules significantly correlated with CH_4_ (Figure 4A), environmental factors such as total nitrogen, total phosphate, organic matter and pH are more likely to drive species interactions among modules that are positively correlated with CH_4_. In addition, Module V was correlated significantly with pH. In previous studies, pH is one of the key factors driving the prokaryotic community in offshore sediments (Yu et al., 2022). This environmental factor mainly affects the prokaryotic community through affecting the structure of microbial cell membrane and availability of soil nutrients (Gabler et al., 2017; Wang et al., 2020). Thus, pH may be an important factor in regulating species interaction to contribute to CH_4_ emission positively. Therefore, under inundation conditions, several environmental factors, especially pH, may tend to contribute positively to methane production and by influencing species interaction.

## 5. Conclusion

The present study revealed the feedback of microbial communities driving methane emissions in coastal soils. Our results indicated that the methane flux increased first and then decreased with the increase of inundation level, and the diversity, community composition and co-occurrence networks of soil prokaryotic, fungal, and cercozoan communities were significantly shifted with different inundation levels. In addition, during the inundation state, methane-associated species and cercozoan community OTUs dominated the species interactions. Environmental factors mainly affected the interaction between species that could promote methane emissions. Overall, simulated inundation triggered some negative correlations between methane-associated species and other microorganisms, and the fungal and cercozoan communities may both play a leading role in regulating methane emission. Moreover, the environmental variables, especially pH, tend to regulate network modules significantly positively related to CH_4._ This study provides a new perspective on microbial cross-trophic relationships for the budget of wetland CH_4_ emissions.

## Data availability statement

The datasets presented in this study can be found in online repositories. The names of the repository/repositories and accession number(s) can be found at: accession number: PRJCA013006 (https://ngdc.cncb.ac.cn/search/?dbId=gsa&q=PRJCA013006).

## Author contributions

LW, MZ, and YD conceived and designed the experiments. LW, YZ, SG, ZhaZ, YW, ZheZ, XD, and XY collected the soil samples and performed the experiments. LW analyzed the data. LW and YD wrote the paper. LW, MZ, XD, KF, SG, YZ, XY, ZhaZ, YW, ZheZ, QZ, BX, GH, and YD contributed substantially to this paper. All authors contributed to the article and approved the submitted version.

## Funding

This project was supported by the National Natural Science Foundation of China (U1906223, 42071126, and 42107136).

## Conflict of interest

The authors declare that the research was conducted in the absence of any commercial or financial relationships that could be construed as a potential conflict of interest.

## Publisher’s note

All claims expressed in this article are solely those of the authors and do not necessarily represent those of their affiliated organizations, or those of the publisher, the editors and the reviewers. Any product that may be evaluated in this article, or claim that may be made by its manufacturer, is not guaranteed or endorsed by the publisher.

## Footnotes

^1^ http://mem.rcees.ac.cn:8080

^2^ http://mem.rcees.ac.cn:8081
