# Supplementary material for: Fungi and cercozoa regulate methane-associated prokaryotes in wetland methane emissions
Source: Front Microbiol. 2023 Jan 6;13:1076610. doi: 10.3389/fmicb.2022.1076610 (PMC9853292; doi:10.3389/fmicb.2022.1076610)

Supplemental Information

Supplemental Table

Table S1 Soil physicochemical properties under low inundation, moderate inundation and high inundation treatments

	TN (mg/kg)	TP (mg/kg)	Ammonium (mg/kg)	Nitrate (mg/kg)	OM(%)	pH	Salinity (ppt)	Oxygen (vol%)	CH_4 flux (n mol CH_4 m^{-2} s^{-1})
IL	1006.4±252.6**a**	523.53±39.17**b**	6.85±2.94**a**	55.66±2.48**a**	1.02±0.37**a**	8.16±0.23**a**	3.71±0.76**a**	21.21±0.03**b**	61.8±45.94**b**
IM	1047.98±313.21**a**	547.08± 101.62**ab**	6.36±3.24**a**	52.56±3.26**b**	0.99±0.24**a**	7.91±0.17**b**	3.83±0.39**a**	21.28±0.02**a**	178.38±41.55**b**
IH	1150.4±325.14**a**	696.83±267.34**a**	8.17±1.69**a**	52.77±1.64**b**	1±0.16**a**	8.03±0.15**ab**	3.5±0.53**a**	21.22±0.01**b**	182.72±54**a**

TN, total nitrogen; TP, total phosphate; OM, organic matter. Low inundation (IL), moderate inundation (IM), high inundation (IH). Values with superscript letters a, b, and c are significantly different across columns ($P < 0.05$).

Table S2. Spearman correlation between gene copy numbers and environmental factors.

	TN (mg/kg)	TP (mg/kg)	Ammonium (mg/kg)	Nitrate (mg/kg)	DOM(%)	pH	Salinity (ppt)	Oxygen (vol%)
mcrA*(*copies/g*)*	0.290	0.153	0.002	-0.189	0.009	-0.372	-0.081	0.369
pmoA*(*copies/g*)*	0.010	-0.208	0.002	-0.172	-0.089	-0.330	0.024	0.629**

The bold numbers are significant values. Asterisks are used to show the significance of p-values. **P <0.01.

Table S3 Dissimilarity tests of microbial communities under low inundation (IL), moderate inundation (IM) and high inundation (IH) treatments.

Dissimilarity based on Bray-Curtis		MRPP		ANOSIM		PERMANOVA	
		r	*P*	r	*P*	r	*P*
Prokaryotes	IL&IM	0.4974	**0.003**	0.2183	**0.037**	2.8518	**0.004**
	IM&IH	0.4854	**0.023**	0.0569	0.218	1.7754	**0.043**
	IL&IH	0.4854	**0.023**	0.0569	0.218	1.7754	**0.043**
Fungi	IL&IM	0.7444	**0.002**	0.4447	**0.001**	2.8851	**0.001**
	IM&IH	0.7677	**0.002**	0.3029	**0.001**	2.259	**0.001**
	IL&IH	0.7191	**0.002**	0.8600	**0.001**	4.7370	**0.001**
Cercozoa	IL&IM	0.5537	0.121	0.0303	0.289	1.5097	0.141
	IM&IH	0.5912	0.093	0.1407	0.062	2.0082	0.085
	IL&IH	0.5746	**0.037**	0.2113	**0.047**	2.8633	**0.023**
Methanotrophs	IL&IM	0.3034	0.838	-0.0458	0.642	0.1640	0.685
	IM&IH	0.2581	0.917	-0.1070	0.993	0.3585	0.577
	IL&IH	0.2488	0.463	-0.0444	0.663	1.1412	0.308
Methanogens	IL&IM	0.6613	**0.032**	0.0287	0.359	1.7638	**0.043**
	IM&IH	0.6902	0.121	0.0570	0.193	1.5742	0.086
	IL&IH	0.6441	0.203	0.0404	0.255	1.3598	0.207

The bold numbers are significant values based on one-way ANOVA.

Table S4. Empirical and randomized molecular ecology network (MENs) properties of intra-trophic networks

		Empirical networks									Random networks		
		**Network size/nodes number**	**Connectivity/ total links**	**Average connectivity**	**Average clustering coefficient**	**Average path distance(GD)**	**Connectance**	**Module number**	**Modularity**	**Vulnerability**	**Average clustering coefficient (avgCC)**	**Average path distance (GD)**	**Modularity**
**Prokaryotes**	IL	1617	5157	6.381	0.643 [a]	1.996 [b]	1.000	28	0.602 [c]	0.055	0.124 ± 0.005	1.996±0	0.326± 0.003
	IM	1253	2737	4.369	0.270 [a]	5.734 [b]	0.506	159	0.721 [c]	0.045	0.009 ± 0.002	4.081± 0.031	0.473± 0.004
	IH	1741	2902	3.334	0.268 [a]	7.299 [b]	0.528	205	0.854 [c]	0.077	0.006 ± 0.002	5.107± 0.037	0.598± 0.003
**Fungi**	IL	122	255	4.180	0.357 [a]	4.814 [b]	1.000	9	0.703 [c]	0.085	0.01 ± 0.009	3.479 ± 0.05	0.455± 0.011
	IM	106	134	2.528	0.161 [a]	5.964 [b]	0.496	15	0.795 [c]	0.283	0.006 ± 0.009	4.647 ± 0.205	0.645± 0.015
	IH	113	178	3.150	0.247 [a]	5.968 [b]	0.581	22	0.728 [c]	0.162	0.013 ± 0.01	3.883 ± 0.11	0.543± 0.012
**Cercozoa**	IL	130	148	2.276	0.259 [a]	5.083 [b]	0.1992	24	0.855 [c]	0.158	0.003± 0.006	6.075 ± 0.312	0.714± 0.013
	IM	98	132	2.694	0.184 [a]	6.412 [b]	0.651	17	0.696 [c]	0.098	0.005 ± 0.009	4.35 ± 0.164	0.607± 0.014
	IH	152	173	2.276	0.173 [a]	7.421 [b]	0.446	24	0.842 [c]	0.281	0.001 ± 0.004	6.349 ± 0.327	0.729± 0.013

Abbreviation:

a Pairwise comparisons were made among the IL, IM, IH groups. Significant difference ($p < .0001$) in average clustering coefficient in prokaryotic, fungal, and cercozoan networks through pairwise comparisons were made among the IL, IM, IH groups based on Student's t test.

b Pairwise comparisons were made among the IL, IM, IH groups. Significant difference ($p < .0001$) in average path distance in prokaryotic, fungal, and cercozoan networks through pairwise comparisons were made among the IL, IM, IH groups based on Student's t test.

c Pairwise comparisons were made among the IL, IM, IH groups. Significant difference ($p < .0001$) in modularity in prokaryotic, fungal, and cercozoan networks through pairwise comparisons were made among the IL, IM, IH groups based on Student's t test.

Table S5. The OTU composition of modules significantly correlated with CH_4 in the interdomain network.

	No.module	Prokaryote[a]	Fungi[a]	Cercozoa[a]	Methanotrophs[a]	Methanogens[a]	Module hub[b]	Annotation
Negative	**Module I**	7(7)	-	-	-	-	-	-
	**Module II**	59(59)	-	-	-	-	OTU_124(B)	Proteobacteria（Phylum)/Methyloceanibacter(Order)
	**Module III**	8(8)	-	-	-	-	-	-
	**Module IV**	8(8)	-	-	-	-	-	-
Positive	**Module V**	-	54(149)	72(149)	10(149)	13(149)	OTU_6979(MC)<br>OTU_8295(MC)<br>OTU_5563(MU)	Euryarchaeota(Phylum)/Methanomassiliicoccales(Order)<br>Euryarchaeota(Phylum)/Methanomassiliicoccales(Order)<br>Proteobacteria(Phylum)/Methylococcales(Order)
	**Module VIII**	-	11(18)	6(18)	-	1(18)	OTU_1912(C)<br>OTU_7813(MC)	Cercozoa(Phylum)/Unclassified(Order)<br>Euryarchaeota(Phylum)/Methanomicrobiales(Order)
	**Module X**	73(73)	-	-	-	-	OTU_957(B)	Proteobacteria（Phylum)/Unclassified(Order)

Abbreviation:

a. The numbers in brackets represent the total number of OTUs for the module. The numbers outside brackets represent the number of OTUs of a particular kindom in the module.

b. The module hubs of each module and its annotation, the name of the OTUs are listed in brackets. B (Prokaryote), F (Fungi), C (Cercozoa), MU (Methanotrophs), MC (Methanogens).

Supplemental Figures

Fig. S1 α diversity (Shannon index, Pielou evenness, Chao1, Observed richness) for prokaryotic, fungal, cercozoan, methanotrophic and methanogenic communities in response to inundation. Soils were exposed to low inundation (IL, n=7), moderate inundation (IM, n=12), and high inundation (IH, n=8). Asterisks indicate a significant divergency (*P <0.05).

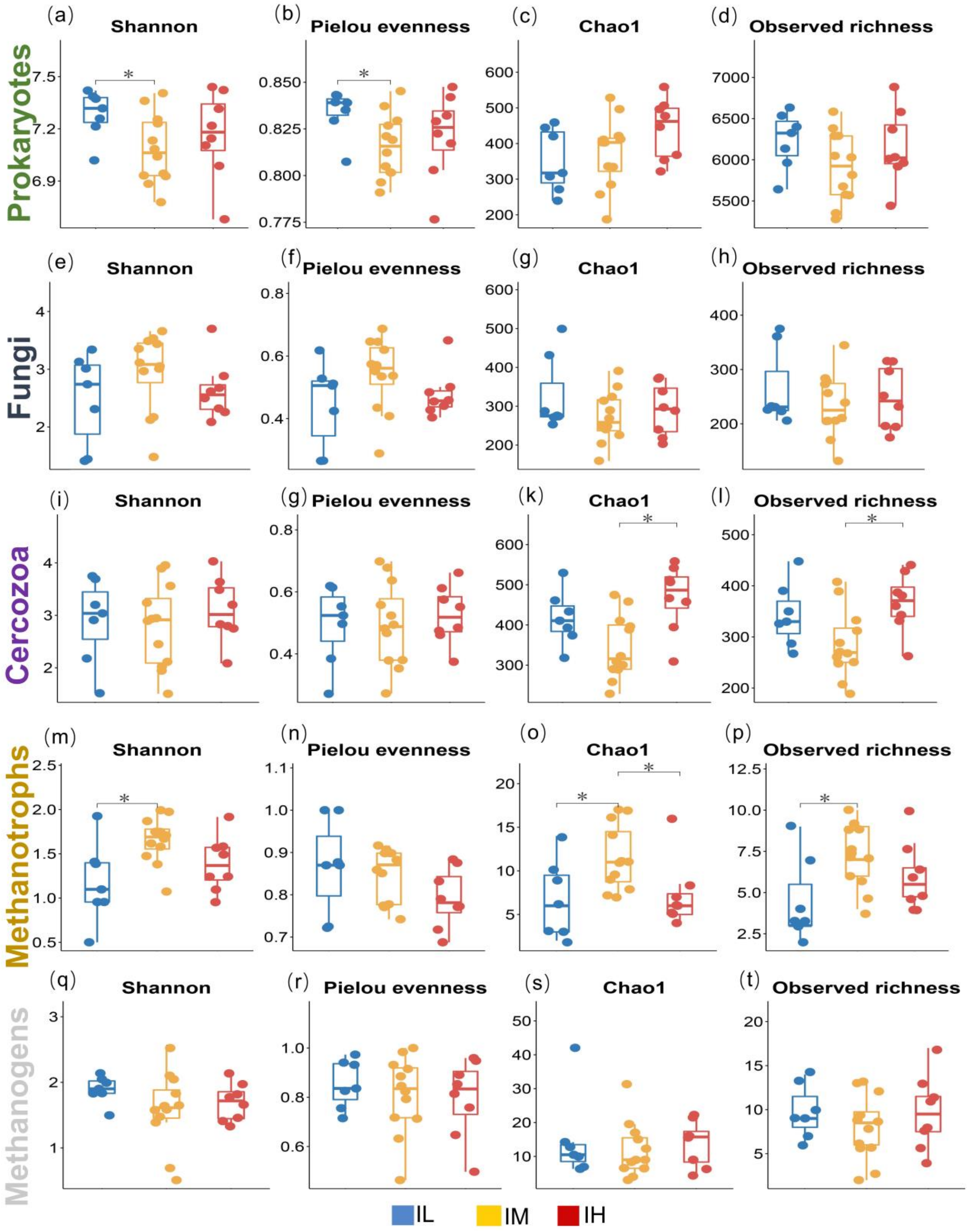

Fig. S2 (a) Principal component Analysis (PCA) conditions of Bray-Curtis for prokaryotic, fungal, cercozoan, methanotrophic and methanogenic communities, in response to inundation. (b) Relative abundance of prokaryotic, fungal, and cercozoan community composition were calculated at the phylum, class, class, order and order level, respectively. Soils were exposed to low inundation (IL, n=7), moderate inundation (IM, n=12) and high inundation (IH, n=8).

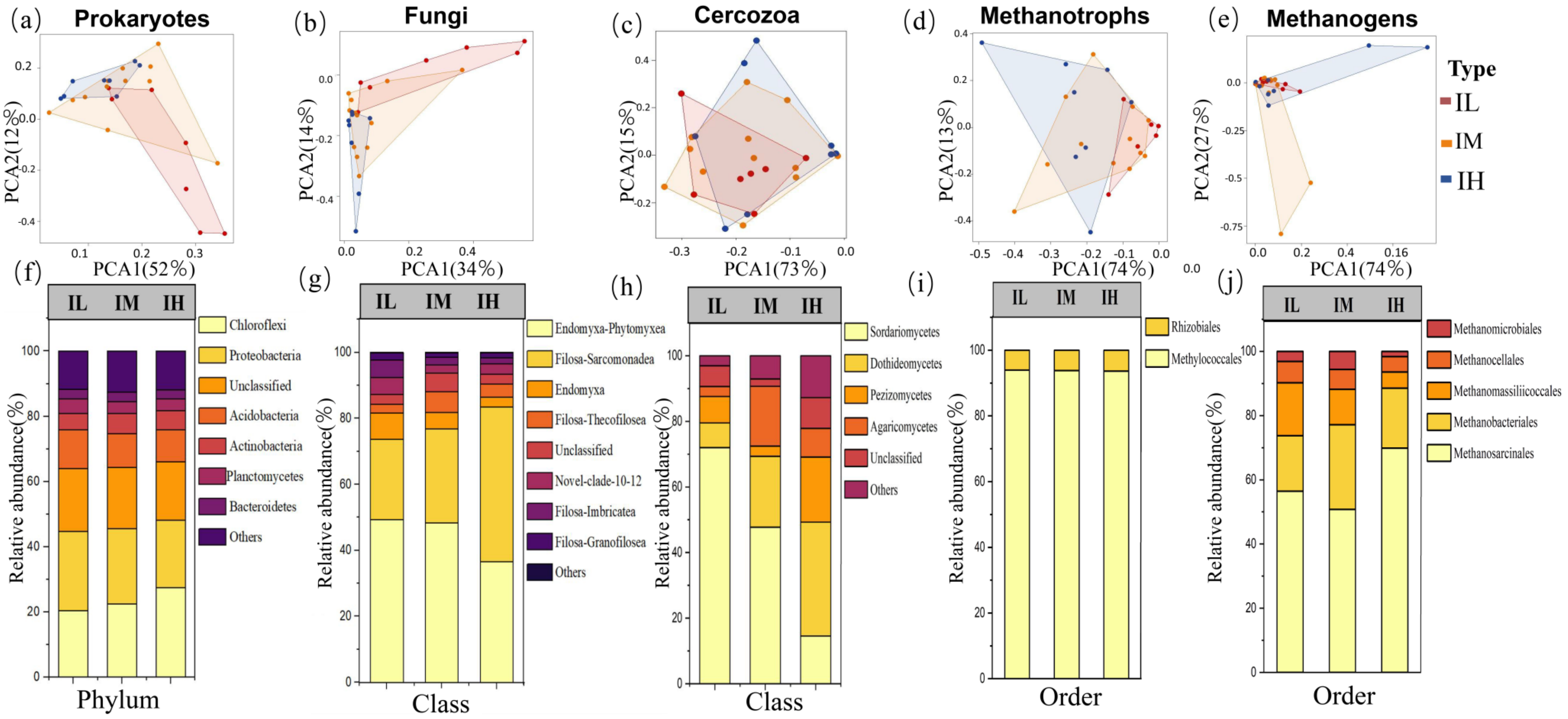

Fig. S3. (a) Functional shifts in prokaryotic communities in IL, IM, IH groups using FARPROTAX. (b) The relative abundance of major methanogenesis pathways.

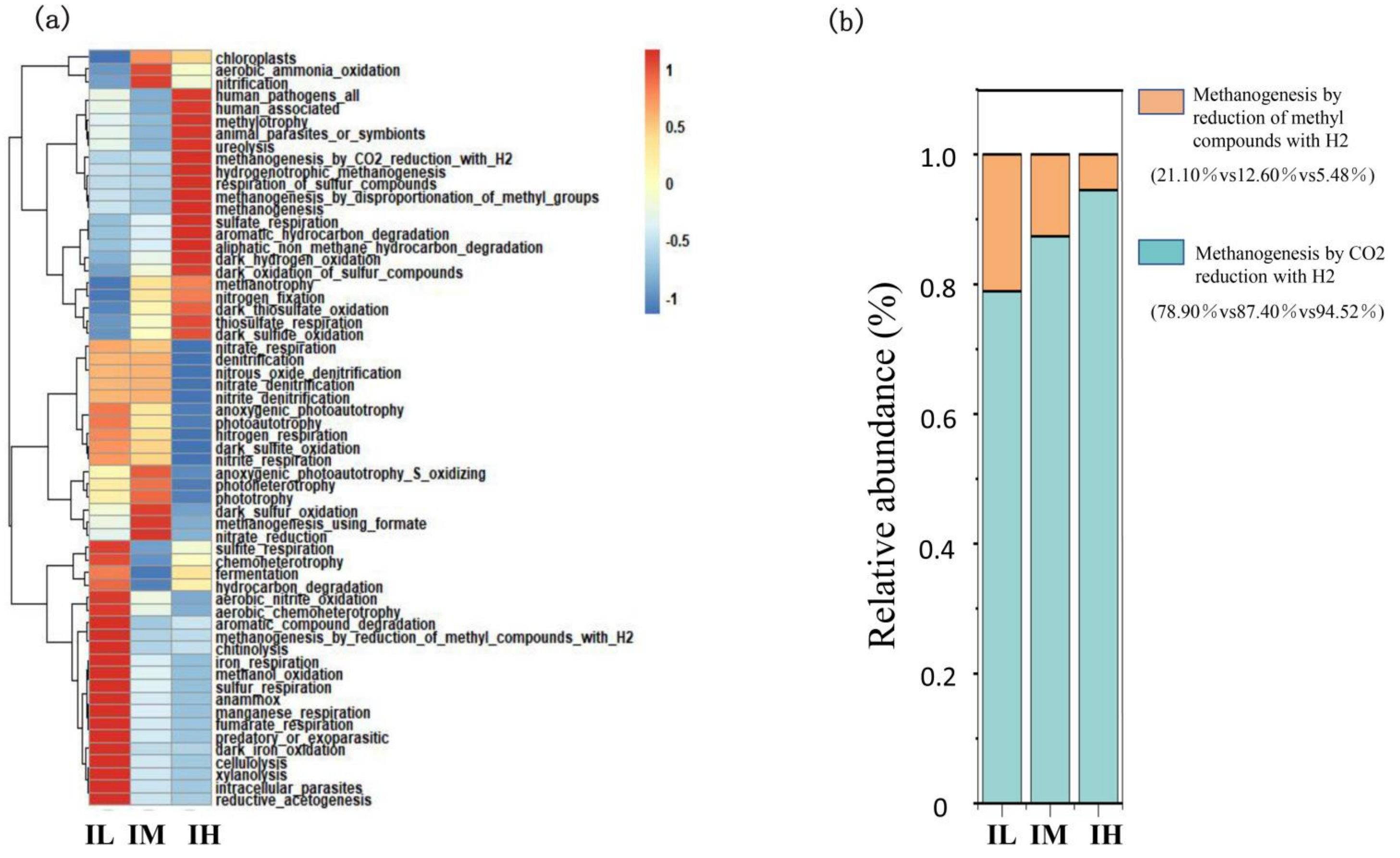

Figure S4. Co-occurrence networks of prokaryotes, fungi, and cercozoa under inundation. Node color indicates taxonomic affiliation. Purple and yellow links represent positive and negative interactions, respectively. Soils were exposed to low inundation (IL, n=7), moderate inundation (IM, n=12) and high inundation (IH, n=8).

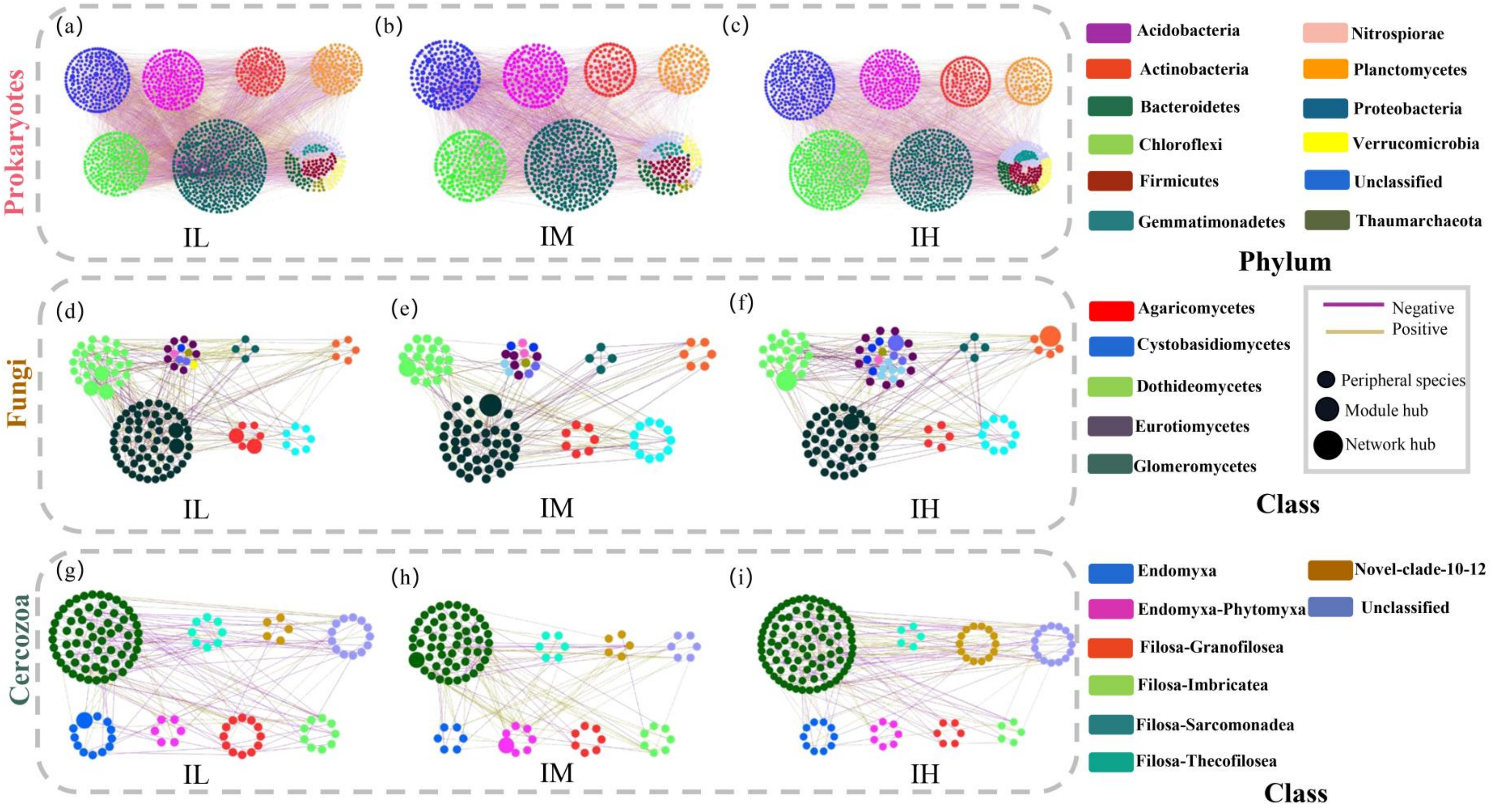

Figure S5. In module V, the four keystone taxa ((a) Methylococcales, (b) Methanomassiliicoccales, (c) Methanomassiliicoccales, (d) cerocozoa)and their connected taxa. Background colors of the surrounding circles represent different communities: fungi (pink), cercozoan (orange), methanotrophic OTUs (green) or methanogenic OTUs (purple). The number in the moderate of every circle represents the number of OTUs.

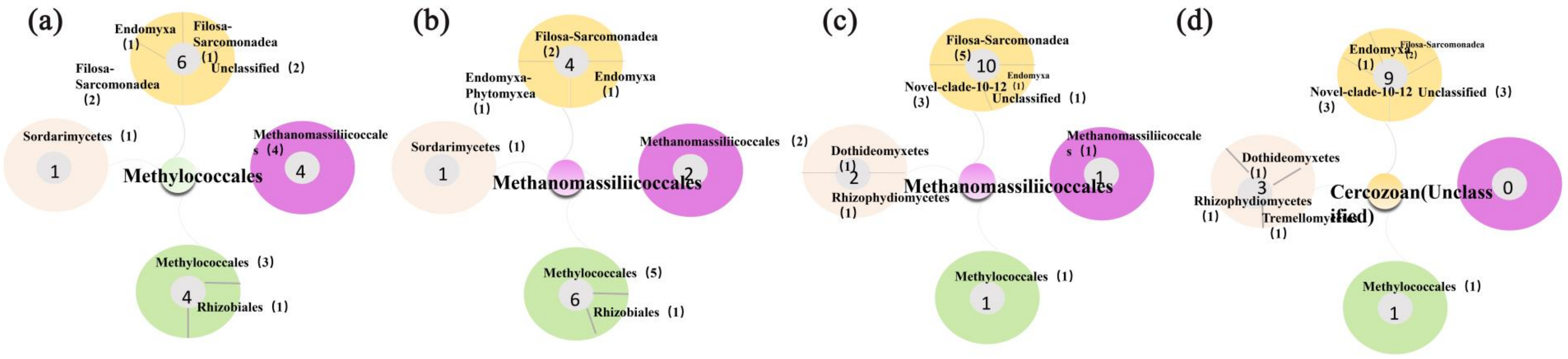

Figure S6. Robustness measured as the proportion of taxa remaining after 50% of the taxa were randomly removed from each of the prokaryotic, fungal, cercozoan empirical MENs.

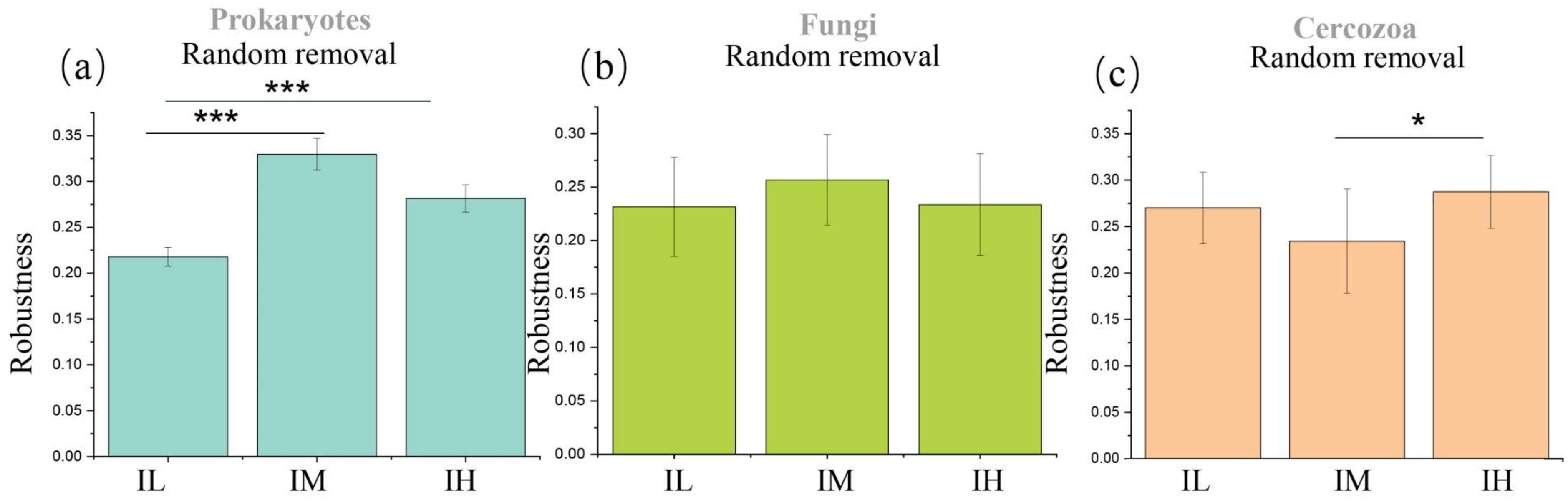

Supplement: Supplementary file 1 [file Data_Sheet_1.pdf]
